# Hospital and Institutional News

**Published:** 1917-01-13

**Authors:** 


### HOSPITAL AND INSTITUTIONAL NEWS.

### SURGICAL SUPPLIES REJECTED BY WAR OFFICE.

We had an opportunity recently of inspecting at a hospital for children a large number of splints of all kinds, bed-tables, bed-rests, cradles, and other articles to the value, probably, of not far short of one hundred pounds, which had been given by one of those voluntary associations which direct the energy of their workers in the direction of the production of surgical supplies. The articles we examined were beautifully made, and formed a most valuable gift to the hospital to which they had been given; in fact the present was so profuse that a number of things were being sorted out to send to a neighbouring hospital, not under military control, where they would be used for wounded soldiers. The fault in the articles, and it is only a fault in a military sense, is that they do not exactly conform with a standard, a bed-table, for instance, being a little out of the regulation pattern or an inch or so too short or too long. For those reasons the goods had been rejected by the military authorities. Standardisation is, no doubt, a very necessary condition with the War Office in matters of this kind, but it is good to think that it did not in this case at least result in utter waste; and the good-natured people who gave so freely of their time and energy will be glad to know their labours are appreciated and their handiwork usefully employed.

### A FINE WAR GIFT FROM NEW YORK.

Captain J. C. Ganzoni, M.P., has announced that Mr. Manuel Rionda, of Wall Street, New York, has authorised the distribution of £8,000 amongst the " sufferers in the good cause defended by the British and Allied nations." Mr. Rionda's generous gift has been distributed, presumably at Captain Ganzoni's instance, among about twenty war charities. One thousand pounds apiece has been given to the British Red Cross Society, the French Red Cross, and the Russian Red Cross. The Italian Red Cross receives £600, the Belgian Red Cross £500; while £200 apiece goes to the Serbian Red Cross, the New Zealand Red Cross, the Australian Red Cross, and the Canadian Red Cross. The Rumanian and South African Red Cross Societies get £100 each. The Russian Prisoners of War Fund takes £400, and St. Dunstan's Hospital for Blinded Soldiers and Sailors comes off well with £700. The Committee for Relief in Belgium is awarded £200, and half that sum is allotted to the War Relief Fund for British Prisoners. Three hundred pounds each is given to the Central Prisoners of War Fund, the Officers' Families Fund, the Fourth Destroyer Flotilla Fund, Lord Roberts' Workshops, and the Navy Employment Agency.

### NEW RULES FOR STREET COLLECTIONS.

The new regulations, published in the *London Gazette* of January 5, made by the Home Secretary to regulate street collections and sales in London for charitable purposes, will have the effect of adding to the comfort of the public and encouraging well-established and respectable charities which are anxious to " play the game " in their efforts to raise funds. The dubious person who used to shake a box at theatre queues, ostensibly collecting for some mysterious Children's Homes or Dinner Fund, the management of which generally cost 75 per cent. of the takings, will now disappear, as no vendor or collector is allowed to receive payment or reward. Other excellent rules are that collectors must be over the age of eighteen, and animals are not allowed to be used for purposes of collecting. A permit to raise money in the manner regulated must be obtained from the Commissioner of Police, who in his decisions will be guided by an advisory committee appointed by himself; we trust that the names of the persons serving on this committee will be published, as this information will be of great interest to those engaged in charitable work. There are several rules which should ensure that the general public, while afforded every opportunity to give, shall not be pestered, and, although there will still remain a great many people who are of opinion that street collections in any form are inadvisable, the general feeling will be that the new regulations are a step in the right direction.

### PROPOSED WAR HOSPITAL FOR SUNDERLAND.

The War Office, that is to say the head of the Army Medical Department, has suggested the erection of a military hospital of 520 beds in Sunderland, at an estimated cost of £30,000; and the mayor of the town has called a meeting of the citizens to consider the proposal. Sir Alfred Keogh has visited the town in connection with the proposal, and has explained to the mayor and other leading townsmen that the present scattering of the wounded in a variety of small hospitals in Sunderland is wasteful both of money and of doctors; and that a war hospital, such as is suggested, under military auspices would be of great advantage to the nation. Sir Berkeley Moynihan, consulting surgeon to the Northern Command, is also enthusiastically advocating the proposed new hospital, of which the people of Sunderland can, we feel sure, be trusted to understand the advantage and necessity. It is designed largely of wood construction, of a single storey, with a main corridor and wards on both sides of it. All the convoys of sick and wounded would be taken direct to this hospital from the station, and dealt with there; and the local hospitals would then be used for transferring those cases from the central hospital as might be regarded as most suitable for such removal. The scheme is apparently only one of several similar projected military hospitals in the large towns.

### THE CHARLES DILKE MEMORIAL SCHEME.

The committee in charge of the proposed hospital in memory of the late Sir Charles Dilke has accomplished the minor half of its purpose. A central site has been obtained at Yew Tree Brake, near Cinderford, Gloucestershire, and the hospital when built will accommodate, according to the present scheme, eight patients, with a staff of three: matron, nurse, and an assistant, presumably a probationer. To this end a sum of £2,568 has been subscribed, together with £500 from the Inglis Charitable Bequest, and £1,000 as an instalment from an anonymous donor. The Crown, which is the chief landed proprietor of the Forest of Dean, has promised £500 if the scheme is approved, plus £25 a year for its maintenance. These are enough to show that the scheme is being pressed forward so far as the circumstances of the time permit, and that the memorial scheme is justifying its inception, which has not always been the case with such schemes even when they have had to supply a genuine need for hospital accommodation.

### WHERE ARE THE HOSPITAL CHAPLAINS?

Not long ago The Hospital published a Note upon the anomalous position of the chaplains in many of the war hospitals. The lesson then enforced has not been learnt, if we may judge from some recent correspondence in the *Church Times*, where a writer, describing herself as a hospital nurse, compared the conduct of the chaplains of the Church of England with that of their Roman or Nonconformist brethren, to the disadvantage of the English Church. She described the attendance of the Church of England chaplains as "*nil* or most superficial," and said that owing to their neglect the patients and staff were turning "in painful bewilderment" to the most paternal of the Churches. Her criticism was enforced by the statement that there was no shortage of Anglican chaplains. She stated that there were "five or six," but that they held aloof "in foolish stiffness and tactlessness," or were "conspicuous by their absence." This amounts to a serious charge, and we hope that the name of this hospital may be published so that the Archbishop of Canterbury and the Chaplain-General of the Forces may be moved to define the precise duties of the chaplains, and to do away with the anomaly, which we have previously noticed, whereby a Church of England chaplain is either supposed to take charge of the men in the recreation huts, as a sort of good fellow, or else has no other work to do.

### THE VOLUNTARY SYSTEM AND EXPERIMENTS ON PATIENTS.

A curious discussion lately took place at a meeting of the Huddersfield Insurance Committee, when Mr. S. Lockwood moved that infirmaries and public hospitals should be withdrawn from private management and placed under municipal control. The resolution, which received only five votes, was lost by a big majority. But it is curious to note that in support of his proposal Mr. Lockwood urged that the establishment of medical schools in connection with hospitals was not favoured by the poor, who objected to being subjected to experiments, and, further, that medical men, by joining honorary medical staffs, practised gratis on the poor in order to be able to charge high fees to the wealthy. Soon after this one doctor left the meeting. On the protest of another, and the remark of a third medical man that the statements were unproved, the chairman, Mr. W. Ramsden, declared that some of them were justified by the findings of the House of Lords' Committee in reference to the metropolitan hospitals. The point, however, which was made by no medical man or anyone else present in answer to Mr. Lockwood, is simply that, even if all his charges had been proved to the hilt, municipal or State control gives absolutely no guarantee that the alleged experiments now suggested would cease. The evidence of fact shows that they would probably be increased. Before the war—we take the liberty of emphasising the date—The Hospital published a series of articles on German hospitals. These are under State control, and, in spite of many merits, our criticism fixed on two facts which we had personally observed at first-hand. The first was that every patient was merely regarded as a case; and the second was that patients were wholly at the mercy of the professors, who made a habit of experimenting upon them even when the object of the experiment had no relation at all to their disease. We commend these facts to Mr. Lockwood, and ask him whether he had better not choose some other ground for the municipalisation which he favours.

### A COMPOSITE MEDICAL STAFF.

Bethnal Green Guardians have prepared a scheme for settling the junior medical staff problem and the temporary arrangements made in consequence of the accident to the late medical superintendent, and to tide over the time that would elapse before the appointment of a permanent medical superintendent. A report of the committee expresses the opinion that the circumstances are now such as to require modification in those arrangements, in order to bring them more into line with the conditions prevailing in similar institutions. The junior medical staff now consists of the following:—Dr. Hamaide (Belgian), at £350 per annum, with residential allowances; Dr. Kapoor (British subject), and Dr. Mersch (Belgian), each at £330 per annum, with residential allowances. The salary assigned to each of these posts was £280, with residential allowances; but in August 1916 Dr. Hamaide's salary was increased, without any application from him, to £350 per annum; whilst Dr. Mersch accepted his post at £280 on July 18, 1916, and the Board increased it to £330 before he commenced duties, as a temporary measure, in view of the vacancy at the time in the post of medical superintendent. The salaries assigned by Hackney for similar posts are £250, with residential allowances; at Islington £240 and £220 respectively; whilst Westminster is now advertising a similar post at £250, with the usual residential allowances. The report therefore suggests that the salaries to be assigned to these posts be at the rate of £250 per annum, rising after six months' service to £300, with the usual residential allowances, and that the existing officers be retained on this scale if they so desire. Under this arrangement Dr. Hamaide, having already served more than six months, will be paid on the maximum scale forthwith; Dr. Kapoor will reach the maximum on January 24, and Dr. Mersch on February 12.

### A DISPUTE ABOUT TERMS AT A RED CROSS HOSPITAL.

DIFFERENCES which have arisen between the Merthyr Guardians and the management committee of the Merthyr and Aberdare Red Cross Society have resulted in the latter body refusing to sign the draft supplemental agreement respecting the Red Cross hospital premises, which belong to the Guardians. The Red Cross Society consider that the agreement is "altogether unfair" to them, and complain that, in addition to keeping the boilers, plant, and machinery in repair, they have to contribute 75 per cent. of the engineer's salary and the whole of the stokers' wages. The Guardians have decided to adhere to these terms—which, they say, have already been agreed to by the Red Cross Society—and, in the event of the Society persisting in its refusal to sign the agreement, to ask the Local Government Board to arbitrate.

### "WITHOUT EXPENSE AND WITHOUT PUBLICITY."

AN important alteration in the rules of a hospital has been made whereby the ancient prohibition under which cases of venereal disease were not admitted has been revoked. This is at the Great Yarmouth General Hospital, where the next step is for the committee to form an agreement with the municipal authorities. In supporting the revocation of the rule, Dr. Shaw said that he hoped that the public would understand that anyone suffering from venereal disease could obtain treatment at the hospital without expense and without publicity. Yarmouth Hospital now falls into line with other institutions which are lending their co-operation to the scheme, of which, in every centre, we hope that the public will take the fullest advantage.

### DEATH OF A LEICESTER BENEFACTOR.

BY the death of Mr. J. A. Corah, J.P., the town of Leicester loses one of its most prominent citizens and benefactors, and the Leicester Royal Infirmary an ardent and enthusiastic administrator. Mr. Corah was the senior partner of the well-known firm of N. Corah and Sons, and the brother of Mr. Alfred Corah, J.P. (the High Sheriff of Leicester and the recently elected Treasurer of the infirmary). He was a member of the board of this institution, and also of the house committee, and both he and his firm were generous benefactors to the institution. Within a few weeks of his death he generously undertook the provision of a recreation room for wounded soldiers at the infirmary, which the board decided to name after him the "J. A. Corah" Recreation Room. His generosity and kindliness of heart were one of the inspirations which induced his workpeople many years ago to introduce systematic weekly contributions for the town's charities, as a result of which yearly sums amounting to about £700 have been distributed for several years. The success of this scheme was largely responsible for the introduction of weekly collections in all the workshops of the town and county under the organisation of the Leicester and County Saturday Hospital Society. Mr. Corah was a Past-Master of the Framework Knitters' Guild, in which he took a great interest. It was during his term as Master that the Guild decided in 1905 to remove the almshouses from Shoreditch to Leicester under a scheme approved by the Charity Commissioners. The financial arrangements largely devolved upon Mr. Corah, who, with enthusiastic energy and personal generosity, was completely successful in raising the large sum required for the building of new almshouses at Oadby, on the outskirts of Leicester. The foundation-stone of these new homes was laid by Sir William Treloar, the then Lord Mayor of London.

### THE FIRST ALLOCATION OF MISS SCHAW'S BEQUESTS.

A WEEK ago the allocation by the trustees of the late Miss Marjory Shanks Schaw, of Glasgow, of the large sum of £100,000 to the Glasgow Royal Infirmary was announced in THE HOSPITAL (January 6, 1917; p. 278). Now the full list of the "first allocation" is available, from which phrase it may be assumed that further benefactions will follow in course of time. In all, grants totalling £311,500 have been made from the residue of her estate under the terms of her trust disposition and settlement; this includes the £100,000 already mentioned. The chief remaining grants are £60,000 to the Western Infirmary; £40,000 to the Victoria Infirmary; and £10,000 each to the following five Glasgow institutions—the Royal Samaritan Hospital for Women, the Royal Hospital for Sick Children, the Royal Maternity and Women's Hospital, the Association for the Relief of Incurables, and the Merchants' House. Nearly seventy institutions in all share in this enormous distribution. A series of bequests to Liverpool institutions is also announced from the estate of Mr. John Camenisch, a cotton broker, of Sefton Park, who left rather more than a quarter of a million. He bequeathed £500 each to the Liverpool Royal Infirmary, the Royal Southern Hospital, and the David Lewis Northern Hospital; £300 each to the Liverpool Queen Victoria District Nursing Association and the Samaritan Free Hospital for Women; and £200 each to the Stanley Hospital and the Children's Infirmary.

### EARLY HOSPITAL STATISTICS FOR 1916.

IT is a sure sign of efficiency when the books and records of a hospital are kept so closely up to date that a complete return of in- and out-patient statistics is available on the first day of the New Year. This is the case at the General Infirmary at Leeds, and a full account of the year's work reached us, prepared in an admirably clear manner, on January 2. The average number of patients daily resident was 402, slightly lower than in 1915; and the number of admissions was 8,844, as against

8,872. About 9 per cent. of the admissions were wounded soldiers. The statistics cover also the Ida and Robert Arthington Semi-Convalescent Hospitals at Cookridge, which are owned and maintained by the General Infirmary, and must be most useful in helping a ready circulation of patients through the parent institution. The infirmary dealt with no fewer than 123,650 out-patient attendances during last year, a weekly average of 2,378. The *x*-ray department skiagraphed 2,648 patients, and 2,590 specimens were examined in the pathological department. The gross numbers of patients have been separated to show the use made of each special department. Altogether Mr. Fred J. Bray, the general manager, and his assistants must be congratulated on a businesslike statistical return, which for promptness and thoroughness will take a good deal of beating.

### PANEL PRACTITIONERS AND MEDICAL MOBILISATION.

THE Panel Committee of the London Insurance Committee has had under consideration the resolution passed by the Central Medical War Committee in regard to the "mobilisation" of the medical profession, and has had under consideration the following recommendations of the General Purposes Sub-Committee: (*a*) That it is essential, in order to secure the efficient working of any scheme adopted, that extended powers be conferred upon the Central Medical War Committee . . . and that the *personnel* of the Committee be strengthened by the addition of at least six practitioners actively engaged in general practice, of whom one should be a woman. (*b*) That the recommendations of the Central Medical War Committee with regard to medical attendance on the civil population should be carried into effect, if accepted, by a small Executive Committee appointed from the members of the Central Medical War Committee with lay members appointed by the Director of National Service. (*c*) That a considerably increased number of medical men could be set free for active service abroad without serious interference with the needs of the civilian population if much greater use were in future made of the part-time services of civilian doctors for the treatment of sick and wounded soldiers.

### EXTENSIONS AT THE HERTFORD COUNTY HOSPITAL.

THE buildings of this old-established institution have been recently largely rearranged and extended to meet the ever-growing calls upon the hospital's services. On the ground floor, the *Labour News* states, the old staircase has been removed, and from the front door a corridor has been driven right through to the other side of the building. A new *x*-ray room has been designed, and has been fitted out with the latest models of radiological equipment and apparatus; and several new offices and bathrooms have been added. On the first floor a new operating theatre unit has been constructed, including a new anæsthetising-room with a surgeon's dressing-room and a new sterilising-room. The floors of these rooms are laid with Terrazzo, and those of the corridors with patent jointless flooring. A new oak staircase has been built to the private and public wards on the second floor. Iron emergency staircases have been fixed on the outside of both the east and west aspects of the hospital. In the basement the servants' room has been improved, particularly in regard to illumination; a crockery store and a box-room for the nurses have been provided; and the kitchen has been modernised and equipped with new appliances. A new hot-water system has been installed, in addition to the provision of a domestic supply: each system is heated by a separate furnace.

### THE RISE IN RAILWAY FARES AND ATTENDANCE AT RURAL HEALTH COMMITTEES.

HEALTH authorities in all parts of the country are passing resolutions of protest, addressed to the Government, against the 50 per cent. increase in railway fares. Not a few have made the suggestion that members of public bodies, who are placing their services at the disposal of the community without fee or reward, should not be required to pay the new impost. There is much to be said in support of this contention, though it is hardly likely that the Government will grant the concession. The burden may not be felt so keenly in city and urban areas, where the members of health committees and hospital boards generally reside within easy reach of the place of meeting, but in many rural districts in different parts of the country, where those who belong to public bodies are drawn from widely-scattered constituencies, any increase in the cost of attending the meetings is a distinct handicap to the efficiency and good working of the body. There are members of public bodies in rural Wales, for example, who have to leave their homes almost as soon as dawn breaks in order to arrive in time for committee meetings held at a central meeting-place at ten o'clock in the morning, and they do not reach their homes again until night-time. Where conditions such as these exist it is sometimes found impossible to get the business of the body transacted at all, and public attention was recently drawn to the case of a hospital authority in Wales the business of which had been held up some months because a sufficient number of members to form a quorum could not be gathered together. If another handicap, in the shape of a heavier railway fare, is to be placed upon the members of rural health committees and hospital authorities, it will prove a serious matter.

### MOTOR AMBULANCE SERVICE IN LONDON.

THE Metropolitan Asylums Board have to face a very serious problem owing to the requirements of the Army and the depletion of their staff of motor repairer mechanics. Mr. B. Portman, the chairman of the Ambulance Committee, and his colleagues have done all they could to impress the various Government Departments to allow them to retain the services of ten efficient repairers, but these have now been reduced to seven, with a further possible reduction of this number. The Board cannot purchase any new chassis, and therefore it is all the more imperative that those they now possess should be kept in thorough repair. At the present time 37 per cent. of the ambulances are awaiting repair, and unless something is quickly done there is a likelihood of the Board being entirely without serviceable ambulances in the near future. This would be a serious disaster in case of a serious outbreak of infectious disease in the Metropolis, for which the Board would be in no way to blame, considering the steps they have taken to retain ten competent repairers, and it is to be hoped that their latest endeavour to get the Government Departments to realise the serious consquences that would arise should there be a dearth of ambulances will awaken the officials and the Government to a full sense of the gravity of the situation.

### THE TESTING OF PANEL DISPENSING.

At a recent meeting of the Plymouth Insurance Committee a resolution was moved to the effect that from time to time the clerk shall, on the instructions of a sub-committee consisting of the chairman, vice-chairman, and a panel doctor selected by the chairman from a rota drawn up for the purpose by the Panel Committee, cause prescriptions against the drug fund to be presented to panel chemists for dispensing. The clerk is to follow the method employed under the Sale of Food and Drugs Act, and the ordinary proceedings are to ensue whenever such a test reveals grounds for complaint. This is the first time, to the best of our knowledge, that such precise and definite steps have been authorised for ensuring that panel dispensing shall be effectively supervised under the Food and Drugs Act. Presumably grounds of complaint, or at least of suspicion, about the quality of drugs supplied or about the care with which prescriptions are dispensed have arisen at Plymouth. Whether the two matters have any connection probably local opinion entertains some idea; but at least it is a coincidence, if nothing more, that at the same meeting of the Insurance Committee various resignations of membership of the Pharmaceutical Committee were reported. The proposal was referred by the Insurance Committee to the Medical Benefits Committee for consideration and report.

### ANOTHER LINK WITH FLORENCE NIGHTINGALE AND THE CRIMEA.

Only two months ago we chronicled the death of a doctor who attended Florence Nightingale when she was ill in hospital during the Crimean War (The Hospital, November 4, 1916, p. 79). Now another link has been snapped by the death at the Kingston Victoria Hospital of Charles Levens, who had served as an orderly under Miss Nightingale and held the Crimean and Turkish medals. Levens was passing along a street in Kingston in an invalid chair, which he propelled himself, when a runaway horse overturned it and threw him into the roadway; he had reached the age of eighty-five. Florence Nightingale's death is so recent, comparatively, that it is difficult sometimes to realise that the Crimean War took place sixty-two years ago. There may be some of her patients still living; but there can be but few, if any, now surviving who were associated with her in her work.

### A CAPITAL COMIC VERSE.

We congratulate the editor of the *Gazette* of the 1st Eastern General Hospital on having secured for his December issue (19th) a good comic story in verse. This amusing and clever poem, which is entitled "A Christmas Story," and signed "Decimal Nought," is remarkable not only for its metrical skill (usually very far to seek in such rhymes), but more especially for the criticism which it contains, and which cuts, as criticism should, below the surface. Now that this *Gazette* is coming out only once a month we hope that Mr. Decimal Nought may be encouraged in such leisure as he has to become a regular contributor. This *Gazette* has waited a long time for anyone so good.

### THE ARTISTS AND THE AUTHORS ON WAR SERVICE.

Modern war, which has been chiefly noticeable for finding everybody useful, has given to the artist and the author an opportunity to use their special gifts in a greater degree than in any previous war. Mr. Derwent Wood has been at work on "oral sculpture"; Mr. Muirhead Bone has been instructed to depict the scenes of battle; Mr. Pennell has been enrolled to sketch the munition factories; and, if we may believe the Germans, no single artist has done more to expose their methods than Mr. Raemaekers; while for the enlightenment of the public Mr. Kipling has described the Fleet, and Mr. Hilaire Belloc has probably done more than any other man to steady civilian opinion and to interpret strategy, tactics, and military values to the general public. To this list, by no means complete, we must add in a new connection Mr. John Galsworthy, who, as a result of a visit to California House, where over 200 disabled Belgian soldiers are being taught applied arts and even languages, has offered, it is understood, the family residence, 8 Cambridge Gate, to the Red Cross for a similar purpose. It will be opened at an early date for British undischarged wounded. Art, letters, and institutions happily meet here on common ground.

### SUBSTITUTION OF WOMEN FOR MEN IN INSANE ASYLUMS.

The question of substitution and the further employment of female attendants in lunatic asylums and other kindred institutions is being forced to the front by the Army's demand for more men. The problem is a difficult one for Tribunals to deal with, and one is hardly surprised at these bodies adopting a "wait and see" policy. The point came before a Welsh (Penybont) Tribunal last week, when Dr. Finlay, the Superintendent of the Glamorgan County Asylum, made application for the exemption of eight of his attendants. The result was the passing by the Tribunal of a resolution asking the authorities to facilitate the substitution scheme so that advantage can be taken of it.

### MANCHESTER ROYAL INFIRMARY.

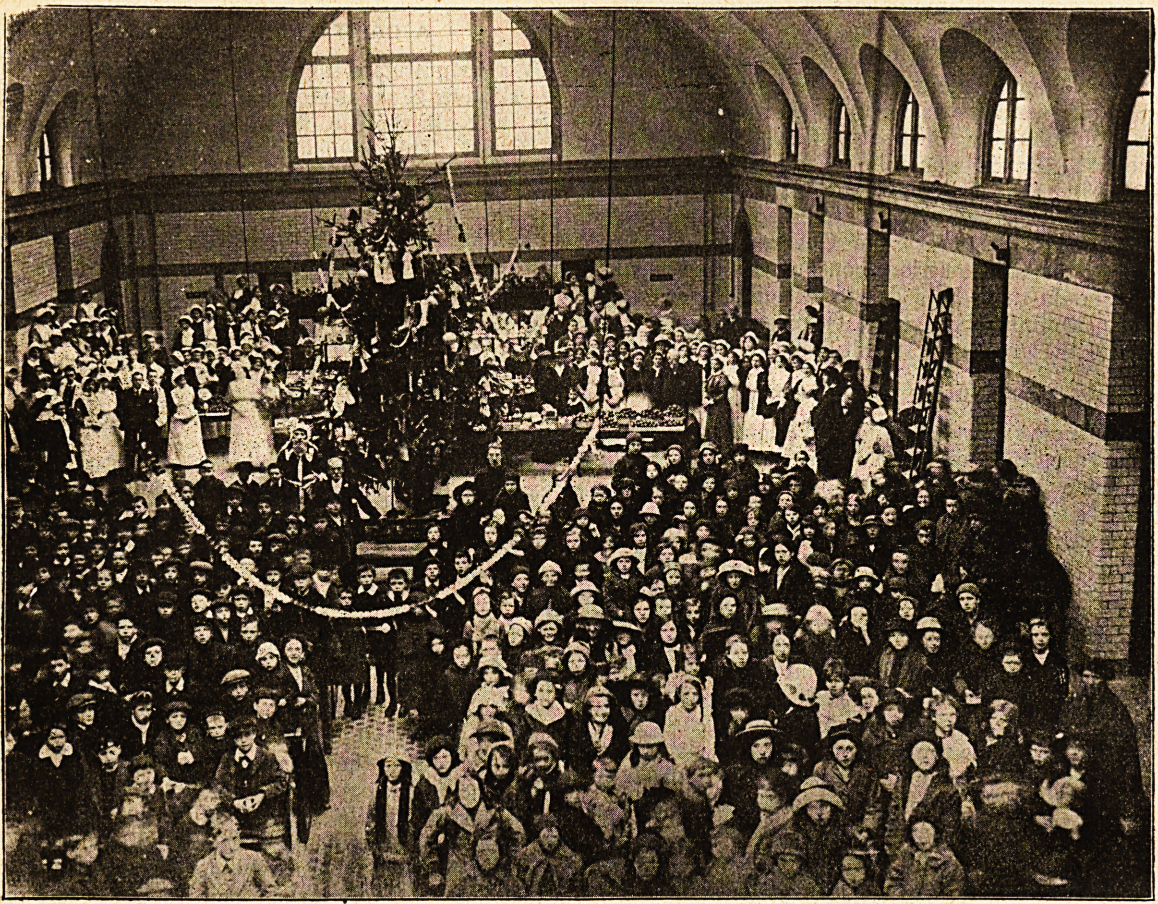

CHRISTMAS IN THE OUT-PATIENT DEPARTMENT.

### ALLOWANCES TO MIDWIVES.

At the meeting of the Hull Corporation Health Committee a letter was read from Sister Edith, hon. secretary of the Hull Midwives' Association, with regard to the maternity scheme in relation to the difficulties experienced by midwives when called in by women after labour has commenced, and it was decided to inform the Association that in the event of a midwife being called in, in an emergency, to attend a patient who is too poor to pay for the services of a midwife, the committee will, on their Medical Officer of Health being satisfied on the matter, pay a fee of 18s. for the attendance at confinement and the following ten days in first cases, and 15s. in other cases, and further that if midwives deem it necessary to call in a medical practitioner in abnormal cases of pregnancy, the fees of the medical practitioner will be paid according to the scale approved.

### THIS WEEK'S DRUG MARKET.

The rise in the cost of spirit—which was anticipated—has naturally led to an advance in the quotations for tinctures and other spirituous preparations. In view of the increasing quantities of alcohol required for the production of munitions, for the Transport and Air Services, and for other purposes directly connected with the war, it seems clear that the supplies available for other purposes will gradually become less and less, and it is probable that the recent further advance in the price of spirit is not the last we shall see before the war is over. Business in the drug market is now quite active again; there are good orders in the market and plenty of inquiries. Price changes, however, have not been numerous. Phenacetin is in rather better supply, and a good business has been done at rather lower prices. Salicylates are also again lower, and a further recession would not come as a surprise. Aspirin also continues to tend downwards in price, but very slowly. Methyl salicylate is rather lower. Phenazone is a trifle dearer. Chloroform has been advanced in price, as also have ethers in consequence of the rise in the cost of spirit.

### TO OUR READERS.

Contributions are specially invited from any of our readers to these columns. They should deal with topical subjects and news. They must be **authenticated for the information of the Editor only. The minimum payment if published is 5s.** There is no hard-and-fast rule as to space, but **notes of about twenty lines in length are preferred.**

